# Circulating S100A12 Levels Are Associated with Progression of Abdominal Aortic Calcification in Hemodialysis Patients

**DOI:** 10.1371/journal.pone.0150145

**Published:** 2016-02-25

**Authors:** Byoung Ho Choi, Han Ro, Eul Sik Jung, Ae Jin Kim, Jae Hyun Chang, Hyun Hee Lee, Wookyung Chung, Ji Yong Jung

**Affiliations:** 1 Division of Nephrology, Department of Internal Medicine, Gachon University of Gil Medical Center, Incheon, Korea; 2 Gachon University School of Medicine, Incheon, Korea; Brigham and Women's Hospital, Harvard Medical School, UNITED STATES

## Abstract

Vascular calcification is an important factor associated with mortality in dialysis patients. Recently, soluble receptor for advanced glycation end product (sRAGE) and extracellular RAGE binding protein S100A12 (EN-RAGE) have been reported to be involved in the process of vascular calcification. Therefore, we investigated whether sRAGE and S100A12 are useful indicators of progression of abdominal aortic calcification in hemodialysis (HD) patients. We analyzed annual changes in vascular calcification score (VCS) for up to 4 years, compared to clinical and biological parameters in 149 HD patients. VCS was assessed annually using plain X-ray images of the lateral lumbar spine. The progression group was defined as patients with an increase in VCS more than 1 point each year on average during the observation period. Time-averaged concentrations were also evaluated to examine the association between biological parameters and changes in VCS. The patients had a mean age of 58.59 ± 12.93 years; 53.7% were male, and 45% were diabetic. The VCS increased in 55 patients; the mean increase was 1.60 ± 2.91 points. In a stepwise multivariate logistic analysis, we found that higher levels of S100A12 were significantly associated with progression of VCS (odds ratio [OR], 2.622; 95% confidence interval [CI], 1.371–5.016; P = 0.004). The relationship between sRAGE and VCS was not statistically significant (OR, 0.644; 95% CI, 0.302–1.374; P = 0.255). Our findings suggest that serum levels of S100A12 are associated with progression of abdominal aortic calcification in HD patients, independent of sRAGE level.

## Introduction

Cardiovascular mortality of patients undergoing dialysis is at least 10-fold higher than that in the general population [1]. Ischemic heart disease, such as acute myocardial infarction, is life threatening. Cardiomyopathy and congestive heart failure, sequelae of cardiovascular events, also have a high mortality rate. The survival rates in dialysis patients tend to decrease after development of cerebrovascular accident and amputation of peripheral vascular disease. Therefore, cardiovascular disease (CVD) is the most important factor that increases the mortality rate of dialysis patients [2].

Vascular calcification is an independent predictor of cardiovascular mortality in hemodialysis (HD) patients [3, 4]. Calcification of vascular cells contributes to atherosclerosis and arteriosclerotic changes in the aorta, coronary arteries, and blood vessels in the brain. The risk factors for vascular calcification in end-stage renal disease (ESRD) have been investigated. Vascular calcification is associated with age, dialysis duration, hypertension, diabetes mellitus, calcium and phosphate ions, parathyroid hormone, vitamin D, and inflammatory cytokines such as interleukin-6 (IL-6) in HD patients [5, 6].

The morbidity and mortality of CVD can be predicted by the vascular calcification scores (VCSs) of abdominal aortic calcific deposits [7]. VCSs can be determined by lateral lumbar spine radiographs, using an aortic calcification severity scoring system. The reproducibility and objectivity of VCS was demonstrated using the 0–24 aortic calcification severity score [8].

Receptor for advanced glycation end products (RAGE) is a multi-ligand member of the immunoglobulin superfamily of cell-surface molecules. Extracellular newly identified RAGE-binding protein (EN-RAGE) is a ligand for RAGE. It is also known as S100 calcium-binding protein A12 (S100A12). The soluble form of RAGE (sRAGE) is a product of both alternative splicing of RAGE mRNA and cleavage of membrane-bound RAGE. S100A12 and sRAGE have recently been found to be associated with vascular calcification[9]. Moreover, S100A12 mediates calcification of vascular smooth muscle cells in vitro and in vivo [10].

A previous cross-sectional study by our group reported that sRAGE has an inverse correlation with vascular calcification in HD patients [11]. Most cross-sectional studies to date have investigated the correlation between vascular calcification and S100A12 and sRAGE, and only few studies have evaluated the progression of vascular calcification in other groups of patients. Therefore, the effects of RAGE on progression of VCS are unknown. Hence, we performed a longitudinal 4-year follow-up observational study and investigated the associations between VCS and S100A12, sRAGE, and other factors at baseline and over time.

## Methods

This study was conducted in accordance with the principle of the Helsinki Declaration and was approved by the institutional review board in Gachon University Gil Medical Center (GBIRB2015-299). Written informed consent was obtained from all participants.

### Study design and setting

This was a prospective and longitudinal observational study of a cohort of patients treated with HD. We evaluated the changes in VCS in the patient population over 4 years and investigated the factors related to the changes.

### Study population

**Fig 1:**
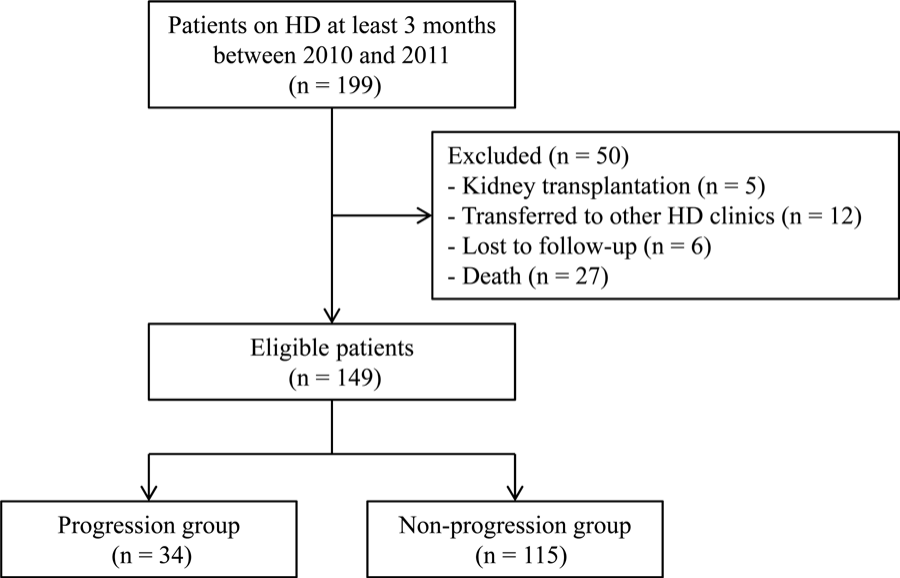
Study flow diagram.

Originally, we enrolled 199 consecutive patients visiting our HD center at Gachon University Gil Medical Center between January 2010 and December 2011. Among the study population, 126 patients were followed up in terms of their clinical, laboratory and lateral lumbar spine X-ray parameters between March 2011 and March 2015. In addition, a further 23 patients were followed up in terms of their clinical data between March 2011 and March 2014. Data of the remaining 50 patients were unavailable because 23 patients were excluded from the study (5 patients received a kidney transplantation, 12 patients were transferred to other dialysis clinics, and 6 patients were lost to follow up) and 27 patients died. Therefore, we ultimately analyzed 149 patients whose data for a period of 3 or 4 years were available (Fig 1).

Patients were eligible for the study if they had undergone HD for more than 1 year and were free of conditions that could affect serum levels of S100A12, sRAGE, and other inflammatory cytokines, such as infection, active liver disease, an underlying malignancy, autoimmune disease, or an indwelling catheter.

### Clinical and laboratory parameters

Immediately prior to mid-week HD sessions, systolic and diastolic blood pressure (BP) were measured and sampling for blood chemistry analyses was carried out after overnight fasting. Blood samples were collected and serum was separated by centrifugation within 1 h of sampling. Isolated serum was stored at below −70°C until it was used for enzyme-linked immunosorbent assay (ELISA). IL-6 levels were determined by ELISA (Human IL-6 Quantikine ELISA Kit, R&D Systems, Minneapolis, MN, USA). The concentrations of sRAGE (Human RAGE Immunoassay; R&D Systems, Minneapolis, USA) and S100A12 (CircuLex S100A12/EN-RAGE ELISA kit, CycLex, Nagano, Japan) in serum were quantified using commercially available ELISA kits according to the instructions of the manufacturer, as described in our previous report [11]. Serum levels of calcium (Ca), phosphorus (P), intact parathyroid hormone (iPTH), 25-hydroxyvitamin D (25D), 1,25-dihydroxyvitamin D (1,25D), high-density lipoprotein cholesterol (HDL-C), low-density lipoprotein cholesterol (LDL-C), triglycerides (TG), albumin, highly sensitive C-reactive protein (hsCRP), and hemoglobin levels in blood were measured using routine laboratory techniques. Biochemical parameters, with the exception of sRAGE, S100A12, and IL-6m, were measured at 1-month intervals during follow-up, and time-averaged values until the end of the study period were calculated. Age, BP, body mass index (BMI), dialysis duration (months), dialysate calcium concentration, single-pool Kt/V (spKt/V), smoking status, history of CVD, and medication history were examined as candidate predictive parameters for VCS.

### Vascular calcification score

VCS was evaluated using plain X-ray images of the lateral lumbar spine in the standing position, as described previously [8]. Anterior and posterior aortic wall calcifications were quantified on a 0–3 grading scale in lumbar vertebrae 1 to 4 according to the severity of calcification: 0, no aortic calcified plaques; 1, small scattered calcified plaques covering less than one third of the longitudinal wall of the aorta; 2, calcified deposit plaques covering one-third or more but less than two-thirds; and 3, calcified deposit plaques covering two-thirds or more. The score of each vertebral segment was determined separately and summed, resulting in a total score of 0 to 24 points. The initial lateral lumbar spine X-ray was performed in March 2011. Further X-rays were performed annually for 3 or 4 years. A single observer read all X-ray images without knowledge of the clinical history of the subjects.

### Statistical analysis

The Kolmogorov–Smirnov test was used to verify the normality of the distribution of continuous variables before further statistical analysis. Natural logarithmic transformation was performed on skewed variables, including S100A12, sRAGE, and IL-6 levels. All variables are reported as means ± standard deviations or medians with interquartile ranges, as appropriate. Otherwise, nominal variables are expressed as percentages. Differences between two groups were evaluated using Student’s t-test for continuous variables and the chi-squared test for nominal variables. Multiple logistic regression analyses were performed to determine the factors related to the progression of VCS, which was defined as an increase of at least 1 point in the VCS per year. All statistical analyses were conducted using the statistical software SPSS version 20.0 (SPSS, Chicago, IL, USA). For all statistical analyses, a two-tailed P < 0.05 was considered to indicate statistical significance.

## Results

### Baseline characteristics of the study patients

The demographic and clinical characteristics of the study patients are shown in Table 1. The patients had a mean age of 58.59 ± 12.93 years; 53.7% were male, 45% were diabetic, and their mean HD duration was 53 (interquartile range, 19–71) months. At baseline, 80.5% of the patients were taking a calcium-based phosphate binder.

**Table 1:** Baseline demographic and clinical characteristics of the study patients.

Variable	Total (n = 149)
Age (years)	58.59 ± 12.93
Gender (male %)	53.7
BMI (kg/m^2^)	22.69 ± 3.40
Cardiovascular	
Diabetes (%)	45
Previous CVD (%)	33.6
Smoking habit (%)	40.9
Blood pressure (mmHg)	
Systolic	143.45 ± 27.26
Diastolic	73.66 ± 14.43
MAP	96.93 ± 16.99
Antihypertensive drugs (%)	
RAS blockers	55.7
Beta-blockers	51
CCB	48.3
Anti-platelet agents (%)	96
Statins (%)	30.9
Ca-based P binder (%)	80.5
Vitamin D analogue (%)	26.8
HD	
Dialysis duration (months) *	53 (19–71)
Dialysate Ca concentration (mmol/L)	1.60 ± 0.21
VCS	
VCS-baseline	5.03 ± 5.89
VCS-last follow up	6.63 ± 6.81
ΔVCS ^†^	1.60 ± 2.91

Abbreviations: BMI, body mass index; CVD, cardiovascular disease; MAP, mean artery pressure; RAS, renin-angiotensin system; CCB, calcium channel blocker; Ca, calcium; VCS, vascular calcification score

* Median, interquartile range

^†^ ΔVCS (difference between last follow-up year VCS and baseline VCS)

The average increase in VCS during the observation period was 1.60 ± 2.91. Table 2 shows the biochemical parameters at enrollment and over time. The mean concentrations of S100A12 and sRAGE were 419.01 ng/mL (interquartile range 26.45–1534.60) and 2410.66 pg/mL (interquartile range 234.22–9837.79), respectively.

**Table 2:** Laboratory parameters of the study patients.

Parameter	At enrollment (n = 149)	Time-averaged values (n = 149)
HDL-C (mg/dL)	41.38 ± 11.97	41.36 ± 11.62
LDL-C (mg/dL)	74.48 ± 25.56	65.01 ± 17.47
TG (mg/dL)	112.22 ± 74.20	108.74 ± 51.86
Albumin (g/L)	3.94 ± 0.42	3.87 ± 0.32
Hemoglobin (g/dL)	11.14 ± 1.18	10.92 ± 0.71
Ca (mg/dL)	8.60 ± 0.80	8.76 ± 0.48
P (mg/dL)	4.73 ± 1.32	4.58 ± 0.88
iPTH (pg/mL)	250.99 ± 310.93	263.69 ± 231.04
hsCRP (mg/dL)	0.34 ± 0.77	1.21 ± 1.31
25D (ng/mL)	12.95 ± 5.72	11.05 ± 3.70
1,25D (pg/mL)	8.96 ± 6.06	17.47 ± 3.97
spKt/V	1.56 ± 0.22	1.59 ± 0.20
S100A12 (ng/mL)*	419.01 ± 331.41	
sRAGE (pg/mL)	2410.66 ± 1651.01	
IL-6 (pg/mL)	2.42 ± 2.27	

Abbreviations: HDL-C, HDL cholesterol; LDL-C, LDL cholesterol; TG, triglyceride; Ca, calcium; P, phosphorus;; iPTH, intact parathyroid hormone; hsCRP, highly sensitive C-reactive protein; 25D, 25-hydroxyvitamin D; 1,25D, 1,25-dihydroxyvitamin D; spKt/V, single pool Kt/V; sRAGE, soluble receptor for advanced glycation end product; IL-6, interleukin 6

* S100A12 level of excluded patients (n = 50) was 388.84 ± 276.36 ng/mL.

### Comparison of the progression and non-progression groups

We defined the progression group as their VCS have been increased more than 1 point each year on average during the investigation period. The progression group included 34 patients and the non-progression group included 115 patients. The clinical characteristics and laboratory parameters of the progression and non-progression groups are shown in Table 3. The patients in the progression group were older (63.15 ± 12.11 vs. 57.24 ± 12.9 years, P = 0.019) and had a higher S100A12 level (log S100A12 6.02 ± 0.85 vs. 5.63 ± 0.83 ng/mL, P = 0.016). The proportion of patients taking a calcium-based phosphate binder was higher in the non-progression group (84.3% vs. 67.6%, P = 0.031). The other parameters, including sRAGE, did not differ significantly between the two groups.

**Table 3:** Comparison of VCS progressors and non-progressors.

Parameter	Progressors (n = 34)	Non-progressors (n = 115)	P
Age (years)	63.15 ± 12.11	57.24 ± 12.9	0.019
Gender (male %)	61.8	51.3	0.283
Diabetes (%)	47.1	44.3	0.780
Cardiovascular			
BMI (kg/m2)	22.19±2.41	22.85±3.64	0.224
Previous CVD (%)	38.2	32.2	0.511
Smoking habit (%)	41.2	40.9	0.974
Antihypertensive drugs (%)			
RAS blockers	55.9	55.7	0.981
Beta-blockers	44.1	53	0.360
CCB	58.8	45.2	0.163
Anti-platelet agents (%)			
Statins (%)	35.3	29.6	0.525
Ca-based P binder (%)	67.6	84.3	0.031
Vitamin D analogue (%)	26.5	27	0.955
HD			
Dialysis duration (months) *	76 (64–108)	76 (61–121)	0.475
spKt/V	1.58 ± 0.20	1.59 ± 0.20	0.670
RAGE			
Log S100A12 (ng/mL)	6.02 ± 0.85	5.63 ± 0.83	0.016
Log sRAGE (pg/mL)	7.54 ± 0.54	7.60 ± 0.67	0.612
Log IL-6 (pg/mL)	0.49 ± 0.69	0.65 ± 0.71	0.238
Laboratory (Time-averaged)			
HDL-C (mg/dL)	40.5 ± 10.92	41.62 ± 11.85	0.624
LDL-C (mg/dL)	67.22 ± 16.53	64.36 ± 17.75	0.404
Albumin (g/L)	3.86 ± 0.33	3.88 ± 0.32	0.849
Hemoglobin (g/dL)	10.88 ± 0.73	10.93 ± 0.7	0.763
Ca (mg/dL)	8.77 ± 0.46	8.75 ± 0.49	0.879
P (mg/dL)	4.62 ± 0.78	4.57 ± 0.91	0.800
iPTH (pg/mL)	239.95 ± 244.33	270.71 ± 227.59	0.497
hsCRP (mg/dL)	1.3 ± 1.5	1.18 ± 1.26	0.643
25D (ng/mL)	11.66 ± 4.67	10.88 ± 3.37	0.280
1,25D (pg/mL)	18.01 ± 4.04	17.31 ± 3.96	0.369

Abbreviations: BMI, body mass index; CVD, cardiovascular disease; MAP, mean artery pressure; RAS, renin-angiotensin system; CCB, calcium channel blocker; Ca, calcium; spKt/V, single pool Kt/V;; sRAGE, soluble receptor for advanced glycation end product; IL-6, interleukin 6; HDL-C, HDL cholesterol; LDL-C, LDL cholesterol; TG, triglyceride; Ca, calcium; P, phosphorus;; iPTH, intact parathyroid hormone; hsCRP, highly sensitive C-reactive protein; 25D, 25-hydroxyvitamin D; 1,25D, 1,25-dihydroxyvitamin D

* Median, interquartile range

### Logistic-regression analysis of associations of subgroup variables with vascular calcification progression

Stepwise multiple logistic regression models were developed to include possible confounding factors (Table 4). Demographic characteristics, cardiovascular risk factors, laboratory parameters, and medication status were included in models 1, 2, 3, and 4, respectively. Log S100A12 level was associated with a higher likelihood of VCS progression (OR 2.622; 95% CI, 1.371–5.016; P = 0.004 in model 4). Log sRAGE was associated with a lower likelihood of VCS progression; however, the odds ratio was not statistically significant (OR 0.644; 95% CI, 0.302–1.374; P = 0.255 in model 4).

**Table 4:** Odds ratios for associations between S100A12 or sRAGE and vascular calcification progression using stepwise logistic regression models.

	Log S100A12 (ng/mL)		Log sRAGE (pg/mL)	
	OR (95% CI)	P	OR (95% CI)	P
Crude	1.828 (1.108–3.018)	0.018	0.856 (0.473–1.551)	0.609
Model 1	1.942 (1.154–3.270)	0.012	0.838 (0.447–1.571)	0.582
Model 2	2.021 (1.193–3.412)	0.009	0.819 (0.427–1.569)	0.546
Model 3	2.280 (1.277–4.070)	0.005	0.756 (0.373–1.532)	0.437
Model 4	2.622 (1.371–5.016)	0.004	0.644 (0.302–1.374)	0.255

Model 1: adjusted for age, sex, and BMI; Model 2: model 1 + adjustment for cardiovascular disease and risk factors (previous CVD, diabetes mellitus, smoking habit, and dialysis duration); Model 3: model 2 + adjustment for laboratory parameters (time-averaged HDL-C, time-averaged LDL-C, time-averaged Ca, time-averaged P, time-averaged iPTH, time-averaged hsCRP, time-averaged 25D, time-averaged 1,25D, time-averaged spKt/V, and log IL-6); Model 4: model 3 + adjustment for medication (RAS blockers, beta-blockers, CCB, statin and Ca-based P binders). OR (95% CI) denotes odds ratio (95% confidence interval).

## Discussion

Our findings showed that S100A12 is a risk factor for progression of abdominal aortic calcification. After adjusting for confounding factors, S100A12 remained significantly related to changes in VCS in HD patients. Therefore, this relationship between S100A12 and aortic calcification aggravation after 4 years of observation demonstrates that S100A12 is associated in the progression of vascular calcification.

An elevated S100A12 level in ESRD patients is correlated with CVD, such as ischemic heart disease, stroke, and peripheral vascular disease. S100A12 was identified as a significant risk factor for CVD in HD patients [12]. In another study, the S100A12 level had a positive independent association with peripheral arterial disease in 152 HD patients [13]. In several other studies, S100A12 was an independent predictor of mortality in HD patients after adjusting for other clinically important parameters [14–16]. However, few studies have examined vascular calcification progression over time as the S100A12 level increases. Wang et al. [17] reported that S100A12 levels and increments thereof were associated with the presence and progression of coronary artery calcification in HD patients. Our results suggest that VCS increased over time according to baseline S100A12 level, similar to the above finding. Although Wang et al. measured coronary artery calcification using electron beam CT, lumbar X-ray is more feasible in clinical practice and has been validated for estimating the progression of VCS. In their study, the rapid progression group was older (as it was in our study), but the albumin level decreased and hsCRP level increased in the rapid progression group, which is not in agreement with our results. Those things may affect the higher portion of rapid progressor group (43%) than our results (23%), although the definitions of rapid progressors were different from us. In addition, we investigated time-averaged laboratory data at monthly intervals during the whole study period to facilitate accurate adjustment for confounding factors. On the other hand, Wang et al. used mean values of baseline laboratory measurements taken during 3 months, which may not reflect those during the entire period of observation.

We previously reported a negative association between sRAGE and the presence of VCS [11]. However, we did not find a statistically significant inverse relationship between sRAGE and vascular calcification progression in the current study. In one study of chronic kidney disease (CKD) patients receiving dialysis, sRAGE was also not related to CVD [16]. However, in other studies, sRAGE was reported to be negatively correlated with aortic calcification and carotid intima-media thickness in dialysis patients [18, 19]. These controversial findings indicate that plasma sRAGE might not protect against vascular damage due to high levels of inflammatory cytokines, including S100A12 and IL-6. In addition, because the sRAGE concentration must be 1000-fold higher than in human plasma for the binding of advanced glycation end products, other biologically active substances related to sRAGE may be involved in downstream signaling [20].

The S100A12/RAGE interaction is related to macrovascular complications [21, 22]. It leads to inflammation-induced damage to the endothelium and migration of smooth muscle cells into blood vessel walls. This results in atherosclerosis and calcification of atherosclerotic plaques [23]. In an animal study, CKD S100A12-transgenic mice had greater vascular medial calcification of the aorta than wild-type littermates not expressing human S100A12 [24]. Moreover, Ling et al. showed that S100A12 might be a therapeutic target to reduce progression of vascular calcification from animal studies utilizing S100A12-binding compounds [25]. On the other hand, sRAGE binds to RAGE ligands and inhibits in a competitive manner the ligand/RAGE interaction to block the adverse effects of RAGE signaling. sRAGE prevents adverse effects of RAGE, such as diabetic atherosclerosis, and protects against atherosclerotic cardiovascular events [26, 27].

In addition to S100A12, S100A8/A9 (S100 calcium-binding protein A8 and A9) is other calgranulin family, which is an activitor of the innate immunity system via Toll-like recetpr 4 [28]. It has been well known as a useful marker for differentiating between inflammatory bowel disease and irritable bowel syndrome. Moreover, it has been recently suggested as a marker for discriminating between pre-renal and intrinsic acute kidney injury [29, 30]. Furthermore, S100A8/A9 is also known to be related with CVD [22]. A case-control study of 237 patients identified a recurrent cardiovascular event, cardiovascular death or myocardial infarction occurred more after acute coronary syndrome in high level S100A8/A9 [31]. S100A8/A9 was detected in calcifying matrix vesicles (MVs) of human atherosclerotic plaque as mediators of vascular calcification [32]. New et al. showed that activated macrophages release MVs with S100A9 complex, which facilitates microcalcification in CKD environment [33]. Taken together, S100A9 may be another factor that contributes to development of vascular calcification in CKD, which is worthy to be investigated in the future.

There were several limitations to this study. First, the S100A12 level was not measured further during the study period. As a result, we could not show the changes in S100A12 level during the observation period. Second, the large proportion of patients was lost because of death or other follow up loss. Third, because our study was an observational study, we were not able to evaluate whether modulation of S100A12 causes a beneficial effect. To overcome these problems, a large-scale and well-organized prospective study of the effects of S100A12 on vascular calcification progression is warranted.

In summary, patients in the progression group were older and had higher S100A12 levels. However, other parameters, including sRAGE level, did not significantly differ between the two groups. After adjusting for confounding factors, the S100A12 level remained significantly related to changes in VCS in HD patients.

In conclusion, we showed that an elevated serum level of S100A12 was an independent determinant of the progression of abdominal aortic calcification determined by lateral lumbar X-ray in HD patients. Further studies are needed to determine the mechanism by which the S100A12 affects vascular calcification and ultimately whether the modulations of S100A12 are likely to mitigate vascular calcification in this subject.
